# Sarcoidosis-Like Granulomatous Lymphadenopathy Mistaken for Neoplastic Disease on Positron Emission Tomography

**DOI:** 10.1177/2324709619892724

**Published:** 2019-12-06

**Authors:** Ali Ammar, Zahia Esber, Sunil Daniel Bali, Garrett Waggoner, Rumi Khan

**Affiliations:** 1 Orlando Health, Orlando, FL, USA; 2 Henry Ford Hospital, Detroit, MI, USA

**Keywords:** World Trade Center, sarcoidosis, granulomatous lung disease, granuloma, PET avid

## Abstract

This is a rare case of sarcoidosis-like granulomatous lymphadenopathy that was initially mistaken for a neoplastic process due to the degree of hypermetabolic changes observed on positron emission tomography (PET) scan. Sarcoid-like granulomatous pulmonary disease is a disorder that has been described in WTC (World Trade Center) Rescue Workers, and also known as post 9/11 sarcoidosis. We present an interesting case of a man who presented with several months of progressive dyspnea and was later discovered to have significant bilateral hilar adenopathy, which was PET avid. Even more interesting, this patient’s symptoms completely resolved without the use of systemic steroids or immune suppressants. This is a condition that requires awareness in order to avoid repeating unnecessary tests of performing interventions on a benign condition that may resolve on its own.

Sarcoidosis is a well-described chronic granulomatous disease with possible genetic and environmental etiological factors that bring on the disease. Sarcoidosis has been described worldwide in all age populations ranging from 20 to 60 years, and in all racial and ethnic groups. It usually develops before the age of 50 years, with the incidence peaking at 20 to 39 years.^1,2^ The immunopathogenesis of sarcoidosis is thought to be related to human leukocyte antigen (HLA) class II and a T-cell response. However, non-HLA pathogenesis processes have also been described.^3^ Diagnosis is aided by endobronchial ultrasound-guided transbronchial needle aspiration of paratracheal, carinal, and peribronchial lymph nodes. Positron emission tomography (PET) scan has sometimes been useful to rule out neoplasm and to identify lymph nodes of interest.

Several cases of sarcoidosis have been described in World Trade Center (WTC) first responders. Based on a case series, symptoms begin approximately 8 years following exposure. However, PET scan results of these patients have not been reported.^4,5^ Moreover, there exists a lack of description for therapy for this process in this population. Some reports note a response to steroids, whereas others do not.

We report the case of a 48-year-old male with history of mild intermittent asthma and mild obstructive sleep apnea who is an ex-firefighter as well as a WTC first responder who presented to a tertiary care center in Orlando, Florida, complaining of left lower quadrant pain. Computed tomography scan performed was consistent with acute diverticulitis; he was treated with several days of intravenous antibiotics; and his abdominal pain resolved. On obtaining further history, the patient reported that he has had progressive dyspnea over the past 6 months but never sought medical attention because he attributed these symptoms to asthma. Hence, a computed tomography scan of thorax was ordered, which revealed an incidental 2-cm left breast mass, diffuse lymphadenopathy, and a right hilar mass encasing the right main stem bronchus (Figure 1). Appropriate outpatient follow-up was arranged, and the patient was discharged.

**Figure 1.**
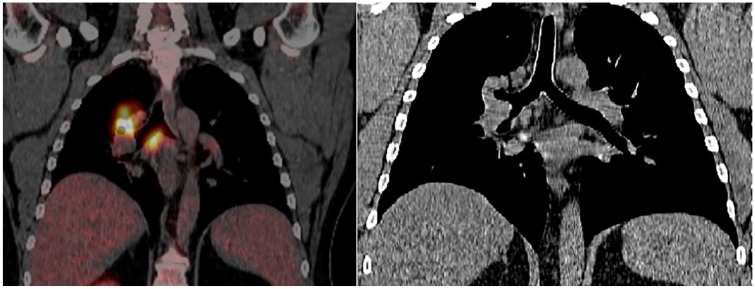
(Left) FDG PET showing highly avid lymph nodes. Right suprahilar lymph node with standardized uptake value (SUV) maximum of 25.4 and paratracheal lymph node with SUV of 18.6. (Right) CT scan of chest showing subcarinal and right hilar lymphadenopathy; the right main stem bronchus shown in mediastinal window.

Due to concern for a neoplastic process, an outpatient PET scan using 18F-fluorodeoxyglucose was ordered and was significant for right pulmonary suprahilar mass demonstrating an standardized uptake value (SUV) maximum of 25.4, and diffuse lymphadenopathy with the highest metabolic activity being 18.6 seen at the level of the subcarina (station 7). Other notable lymphadenopathy was noted in the lower paratracheal area with SUV of 12.3 and right anterior mediastinal adenopathy with SUV of 6.6 (Figure 2).

**Figure 2.**
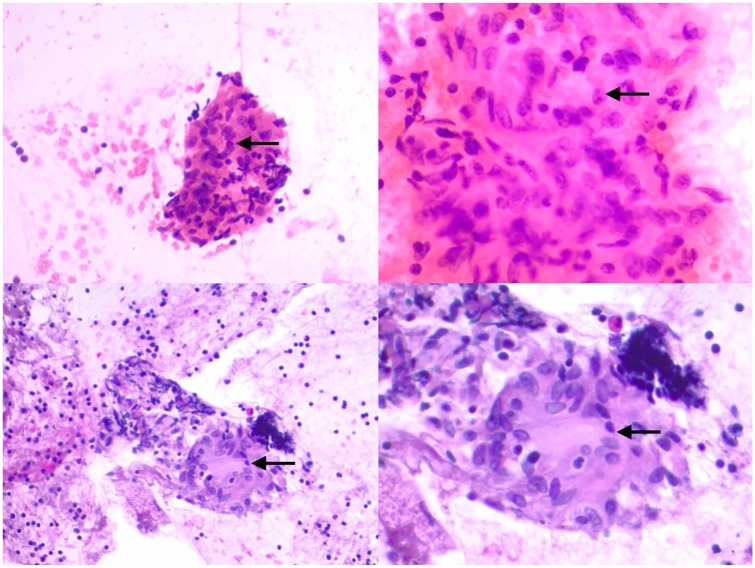
(Top left) Hematoxylin and eosin stain showing noncaseating granulomas at 200× magnification from the peribronchial lymph node (station 10R). Arrow points to area of granuloma. (Top right) Hematoxylin and eosin stain showing noncaseating granulomas from the peribronchial lymph node (station 10R). 400× magnification showing epithelioid histiocytes with footprint-shaped nuclei and abundant eosinophilic cytoplasm. (Bottom left) Hematoxylin and eosin stain prepared in CytoLyt showing noncaseating granulomas at 200× magnification from the subcarinal lymph node (station 7). Arrow points to area of granuloma. (Bottom right) Hematoxylin and eosin stain that was prepared in CytoLyt showing noncaseating granulomas from the subcarinal lymph node (station 10R). 400× magnification showing epithelioid histiocytes with footprint-shaped nuclei.

The oncology team was consulted, and it recommended a tissue biopsy. Endobronchial ultrasound-guided transbronchial needle aspiration was performed at the subcarinal lymph node station level 7 and right peribronchial lymph node (station level 10R). Specimens were collected on formalin and on saline, and sent for cytology and microbiological workup. Bronchoalveolar lavage was also performed in the bilateral upper lobes. Bronchoalveolar lavage and lymph node tissue cultures were negative for infectious etiology. Cytopathology was evaluated at the peribronchial lymph node (station 10R) and subcarinal lymph node (station 7), which were all of samples were consistent with noncaseating granuloma with giant cell reaction (Figure 2). Left breast mass were also sampled and found to be negative for infection and malignancy; it did not show granulomatous disease. Serum C-reactive protein level, rheumatoid factor, ANA, and SPEP/sIFE FLC ratio Ig panel CEA were all negative.

Three months after diagnosis, patient stated that he continued to experience dyspnea and cough that was not like his usual asthma symptoms. Pulmonary function testing was performed, which was consistent with mild intrinsic restrictive lung disease with significant bronchodilator responsiveness by 29% and >200 mL. He was found to have a forced expiratory volume in 1 second/forced vital capacity (FEV1/FVC) ratio of 85, total lung capacity of 65%, FVC of 56%, FEV1 61%, and with diffusion capacity of 65% after correction for hemoglobin.

Despite having a bronchodilator response on pulmonary function tests, his dyspnea no longer clinically responded to short-acting bronchodilators. This clinical decline was attributed to his new diagnosis of sarcoidosis. A trial of prednisone was attempted; however, after only 4 days of prednisone therapy, he began to experience dizziness, headaches, and insomnia, and thus self-terminated the steroid therapy. In an effort to treat his dyspnea, he was started on inhaled long-acting β-agonist and an inhaled corticosteroid (LABA/ICS) and returned to clinic 8 weeks later. On his return to clinic, he stated that his dyspnea, cough, and wheezing had completely resolved, and he had stopped using his LABA/ICS inhaler 2 weeks prior to his visit. He stated that he stopped using the LABA/ICS due to resolution of his symptoms spontaneously. Follow-up chest imaging showed near complete resolution of his hilar lymphadenopathy. At the 3-month follow-up, the patient was completely symptom free, off all inhalers, and having only taken 4 days of prednisone.

This case is quite interesting due to the fact that sarcoidosis usually presents as bilateral hilar adenopathy, particularly, in a lambda pattern. However, we notice that it is predominantly unilateral to the right and involved the subcarinal lymph nodes as well. The majority of WTC-associated sarcoidosis cases do present quite typically, with symmetric hilar and mediastinal lymphadenopathy with mid to upper lung nodules; however, 20% of cases presented in atypical fashion.^6^

In addition, there is a delay in the development of sarcoidosis from the exposure to the clinical manifestations. It is likely this was a slow development of disease. It is also possible that the patient had lymphadenopathy that remained undiagnosed for over a decade and only recently manifested or became activated. Unfortunately, we do not have prior scans to quantify the progression or chronicity of the adenopathy. In one recently published study, it was found that intrathoracic involvement has resolved in up to 45% of WTC-associated sarcoidosis, but perihilar lymphadenopathy persisted in 53% of patients.^4^ We postulate that, given the delay in presentation and diagnosis, the gradual progression of disease state in this individual has lent itself to an abnormal unilateral phenotypic presentation. With regard to this case, it is possible that others with history of exposure could suffer from future progression of disease.

It is possible that 9/11 sarcoidosis is a self-resolving condition, or even more interestingly, it is possible that these rare cases may respond to ICS and may not require systemic steroids. Furthermore, recognition of this condition is imperative among physicians in order to avoid unnecessary oncologic workup for a benign condition. There is more to learn about 9/11 sarcoidosis, and it will be interesting to see what treatment options there may be in the future for these patients.
